# Ki67 Index Changes and Tumor-Infiltrating Lymphocyte Levels Impact the Prognosis of Triple-Negative Breast Cancer Patients With Residual Disease After Neoadjuvant Chemotherapy

**DOI:** 10.3389/fonc.2021.668610

**Published:** 2021-06-21

**Authors:** Yihua Wang, Beige Zong, Yu Yu, Yu Wang, Zhenrong Tang, Rui Chen, Man Huang, Shengchun Liu

**Affiliations:** ^1^ Department of Endocrine and Breast Surgery, The First Affiliated Hospital of Chongqing Medical University, Chongqing, China; ^2^ Department of Pathology, Chongqing Medical University, Chongqing, China; ^3^ Department of Thyroid and Breast Surgery, The Affiliated Hospital of Zunyi Medical University, Zunyi, China

**Keywords:** triple-negative breast cancer, Ki67, tumor-infiltrating lymphocytes, neoadjuvant chemotherapy, residual disease, prognostic factor

## Abstract

**Purpose:**

The aim of this study was to assess the prognostic influence of Ki67 index changes in patients with primary triple-negative breast cancer (TNBC) treated with neoadjuvant chemotherapy (NAC), and to evaluate whether the combination of Ki67 index changes and residual disease (RD) tumor-infiltrating lymphocytes (TILs) provides additional prognostic information for this group.

**Materials and Methods:**

Data from 109 patients with primary TNBC and RD after NAC were analyzed retrospectively. Ki67 changes and RD TIL levels were investigated for associations with recurrence-free survival (RFS) and overall survival (OS) using Kaplan–Meier and Cox analyses.

**Results:**

Ki67 index decreased after NAC in 53 patients (48.6%) and high RD TIL levels (≥30%) were observed in 54 patients (49.5%). In multivariate Cox analyses, no Ki67 decrease status and low RD TIL levels were significantly associated with reduced RFS (hazard ratio (HR): 2.038, 95% confidence interval (CI): 1.135–3.658, P = 0.017; HR: 2.493, 95% CI: 1.335–4.653, P = 0.004), and OS (HR: 2.187, 95% CI: 1.173–4.077, P = 0.014; HR: 2.499, 95% CI: 1.285–4.858, P = 0.007), respectively. Notably, low RD TIL levels were significantly associated with reduced RFS (HR: 3.567, 95% CI: 1.475–8.624, P = 0.005) and reduced OS (HR: 3.873, 95% CI: 1.512–9.918, P = 0.005) in only the no Ki67 decrease group. The differences in 3-year RFS and OS between patients with no Ki67 decrease and low or high RD TIL levels were 24.4% *vs* 79.1% (P = 0.0001) and 33.1% *vs* 87.5% (P = 0.0001), respectively.

**Conclusion:**

Ki67 index changes and RD TIL levels were associated with the prognosis of patients with primary TNBC with RD after NAC. RD TIL levels had greater prognostic significance in the no Ki67 decrease group.

## Introduction

Triple-negative breast cancer (TNBC) is a specific subtype with an aggressive clinical manifestation that accounts for approximately 15–20% of breast cancers. TNBCs tend be a higher clinical stage and are more prone to recurrence and metastasis than other breast cancer subtypes (1). Neoadjuvant chemotherapy (NAC) has become an integral part of the systematic treatment of TNBC. A major advantage of this strategy is the ability to observe the tumor response to chemotherapy regimens before surgery (2). Patients with TNBC who achieve pathological complete response (pCR) after NAC have better prognosis than those who do not reach pCR (3, 4); however, numerous patients with TNBC have residual disease (RD) after NAC, which is associated with a higher risk of relapse and distant metastasis (5, 6). Novel prognostic biomarkers that can stratify these patients will be valuable for making individualized treatment decisions and maximizing therapeutic efficacy in specific patient groups.

Tumor-infiltrating lymphocytes (TILs) are key tumor immune-related factors, which can communicate with the tumor microenvironment and mediate immune responses against the tumor (7, 8). There is currently significant research interest in the prognostic impact of TIL levels in patients with breast cancer. Growing evidence shows that higher pre-treatment TIL levels are associated with better prognosis in patients with breast cancer in both neoadjuvant and adjuvant settings (9–11). Moreover, several studies have evaluated residual lesions in patients with TNBC and RD after NAC and found that high RD TIL levels are associated with better relapse-free survival (RFS) and overall survival (OS) (12, 13). Real-world data from patients with TNBC in our region may provide new information regarding the prognostic significance of RD TIL levels.

Ki67 index is an indicator of malignant proliferation activity, which has been extensively investigated as a prognostic indicator in breast cancer (14). It is established that the Ki67 index in breast cancer changes dynamically after NAC, indicating that tumor proliferation ability may alter following NAC (15, 16). The Ki67 index is closely related to local recurrence and distant metastasis of breast cancer and there is some evidence that a decreased Ki67 index after NAC is associated with favorable clinical outcomes (16–18); however, there have been limited studies on the impact of this biomarker on the prognosis of patients with TNBC and RD.

The primary objective of the present study was to assess the independent prognostic influence of changes in Ki67 index in patients with primary TNBC following NAC. The secondary objective was to evaluate whether the combination of changes in Ki67 index and RD TIL levels provides additional prognostic information for this group.

## Materials and Methods

### Patients and Treatments

**Figure 1:**
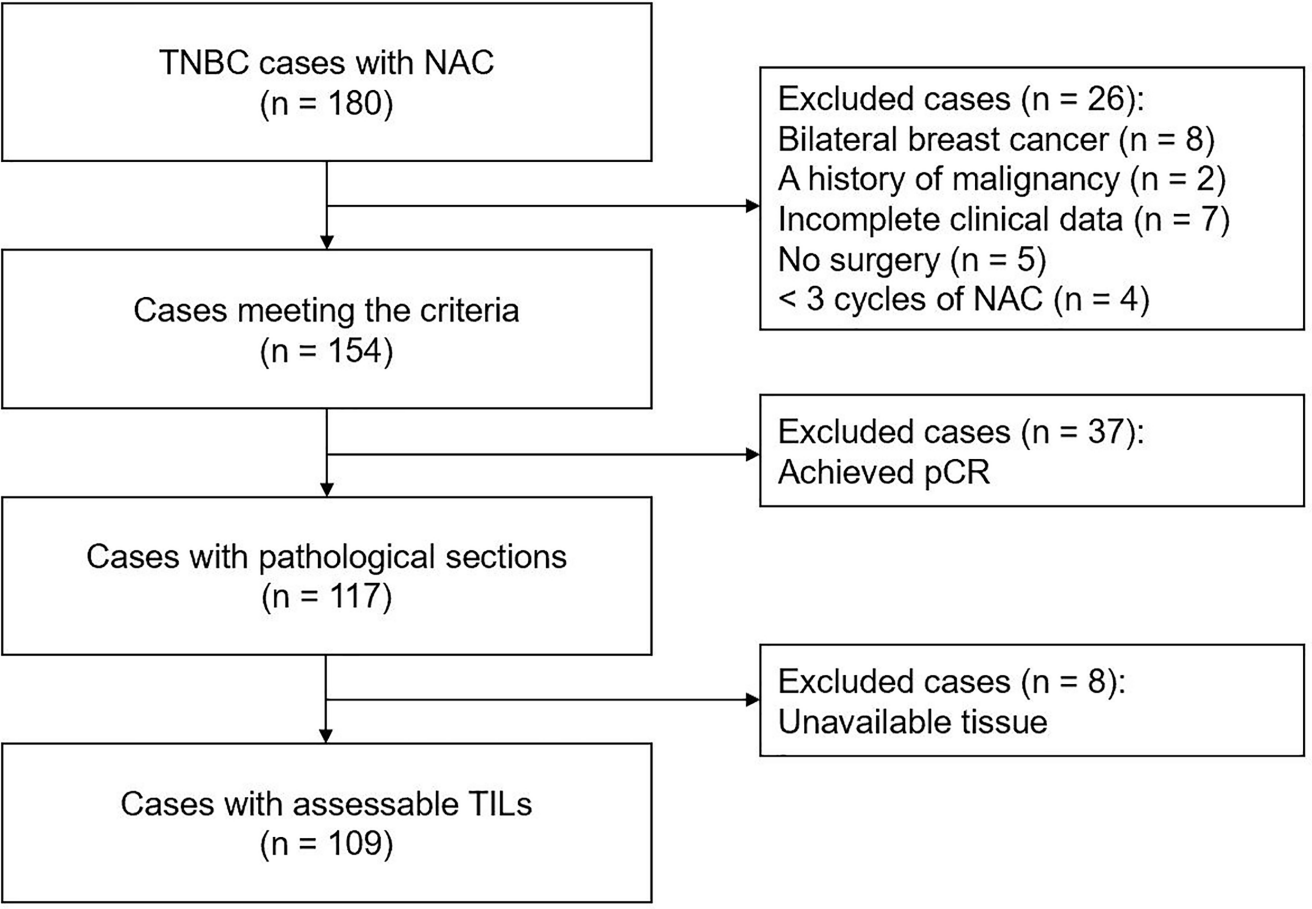
Flowchart of patient selection in present study. TNBC, triple-negative breast cancer; NAC, neoadjuvant chemotherapy; pCR, pathological complete response; TILs, tumor-infiltrating lymphocytes.

In this retrospective study, 180 consecutive female patients with non-metastatic TNBC treated with NAC at the First Affiliated Hospital of Chongqing Medical University between November 2012 and August 2018 were assessed. The exclusion criteria were as follows: (1) patients with previous cancer, concomitant cancer, or bilateral breast cancer; (2) patients who received <three cycles of NAC or did not undergo surgery; (3) patients with incomplete clinical data; (4) patients who achieved pCR after NAC; and (5) patients with unevaluable RD TIL levels or Ki67 index. Finally, 109 patients were included in this study (**Figure 1**). All 109 included patients underwent NAC every 21 days [mean number of cycles: 4 (range, 3–8)]. The majority (90.8%) received an anthracycline plus taxane regimen. Four patients were treated with a taxane-based regimen, and six with an anthracycline-based regimen. Medical records were reviewed to collect clinicopathological data, including age, menopausal status, tumor size, lymph node involvement, histological subtype, histological grade, and surgical procedure.

This research was conducted ethically in accordance with the World Medical Association Declaration of Helsinki and was approved by the Ethics Committee of the First Affiliated Hospital of Chongqing Medical University (No. 2020-59), who deemed that written informed consent was not necessary due to the retrospective nature of this research.

### Histological Evaluation and Immunohistochemistry

All pathological results were re-evaluated independently by two pathologists with no knowledge of patient outcomes. RD molecular subtype was confirmed as TNBC in all participants before inclusion. TNBC was defined as estrogen-receptor (ER), progesterone-receptor (PR), and human epidermal growth factor receptor 2 (HER2) -negative (19). ER and PR status were considered negative if <1% of tumor cells were stained, and HER2 status was considered negative if a score of 0 or 1+ was confirmed by immunohistochemistry, or no HER2/*neu* gene amplification was detected by fluorescence *in situ* hybridization. pCR was defined as the absence of residual invasive tumor lesions in any breast tissue or lymph node (ypT0ypN0 or ypT0/is ypN0) (20).

**Figure 2:**
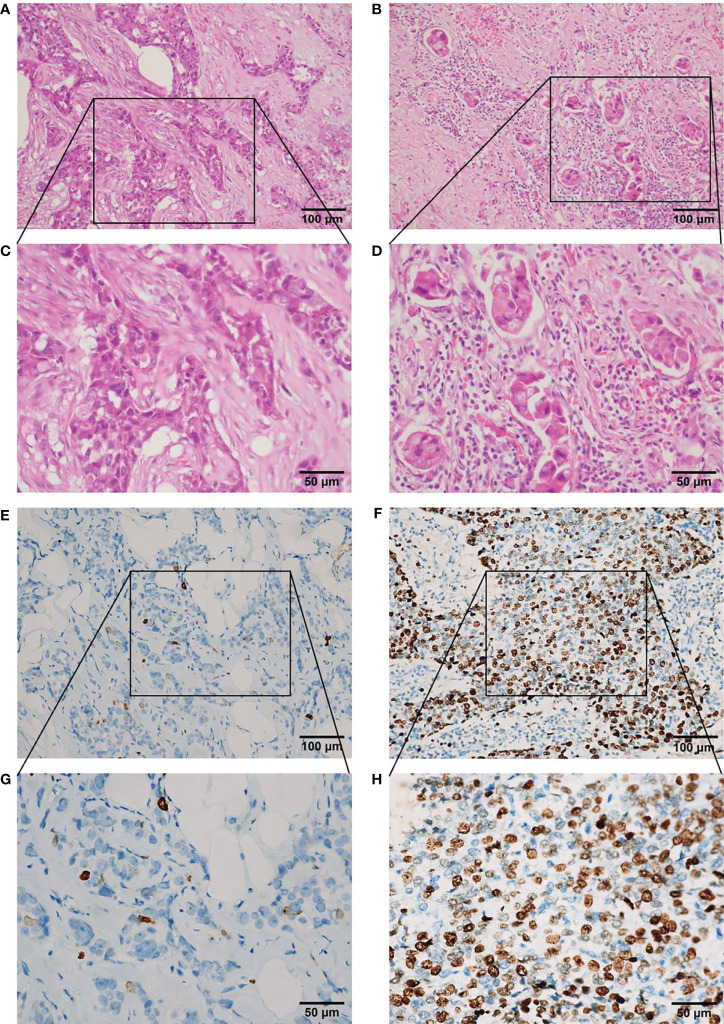
Representative photomicrographs of TILs in hematoxylin and eosin sections and Ki67 index in immunohistochemical sections in residual disease in triple-negative breast cancer after neoadjuvant chemotherapy. **(A)** Low level (<30%) of TILs (×200 magnification). **(B)** High level ≥30%) of TILs (×200 magnification). **(C)** Low level (<30%) of TILs (×400 magnification). **(D)** High level (≥30%) of TILs (**x** 400 magnification). **(E)** Low level (<14%) of Ki67 index (×200 magnification). **(F)** High level (≥14%) of Ki67 index (×200 magnification). **(G)** Low level (<14%) of Ki67 index (×400 magnification). **(H)** High level (≥14%) of Ki67 index (×400 magnification). TILs, tumor-infiltrating lymphocytes.

Regarding the Ki67 index, between 500 and 1,000 cells were counted to calculate the percentage of positive tumor cells in the invasive front of the tumor with nuclear staining, as advised by the International Ki67 in Breast Cancer Working Group (using the Global Scoring method) (21). To evaluate changes in Ki67 after NAC, the Ki67 indices were assessed in biopsy specimens before NAC and surgical specimens after NAC from the same patient. According to the report of Matsubara et al. (22), Ki67 decrease was defined as a decrease in the baseline Ki67 index of >1% after NAC. Histopathological evaluation of the percentage of TILs was conducted using hematoxylin and eosin (H&E)-stained sections from surgical specimens, according to the recommendations of the International TILs Working Group 2014 (23). Briefly, quantification of TILs in the tumor stroma was recorded as the percentage of occupied stromal areas (13). Based on the study of Liu et al. (24), the cut-off value applied for the percentage of TILs was 30% (**Figure 2**).

### Follow-Up

Follow-up investigations, including a clinical examination and a radiological assessment, were performed at regular intervals (3-month intervals in years 1–3, 6-month intervals in years 4–5, and 12-month intervals in years 6–10 after surgery). Detailed information on patients with recurrence, metastasis, or death was accurately recorded. OS and RFS were defined as per the STEEP classification (25). OS was defined as the length of time from the date of tumor diagnosis to the date of death from any cause, or to the date of the last visit. RFS was calculated from the date of surgery to local, regional, or distant recurrence, or to the date of death from any cause. The deadline for follow-up was February 1, 2021.

### Statistical Analysis

All data were analyzed using SPSS statistics software, version 25.0 (SPSS Inc., Chicago, IL, USA). Categorical variables are presented as numbers and percentages and were compared *via* Chi-square and Fisher’s exact tests. The Kaplan–Meier method was used to estimate the distributions of OS and RFS, and the log-rank test was used to compare survival distributions among groups. Univariate Cox proportional hazards models were fit to assess the association between baseline variables and clinical outcomes. Multivariate Cox proportional hazards models were fit to assess the association of each baseline covariate with clinical outcomes, while adjusting for patient and disease characteristics. Results are expressed as hazard ratios (HRs) and 95% confidence intervals (CIs). Statistical significance was defined as a two-sided P value <0.05.

## Results

### Patient Characteristics

A total of 109 TNBC cases with evaluable RD TIL levels were eligible for analysis. Patient baseline characteristics are shown in **Table 1**. Mean age was 47.8 years (range: 20–76 years), 41 patients (37.6%) were postmenopausal and 68 (62.4%) were premenopausal or perimenopausal. Invasive ductal carcinoma constituted the most frequent histopathological subtype (90.8%). Excluding 15 unavailable cases, the most common histological grade was II (53.2%), followed by III (32.1%), and I (0.9%). The majority of patients (90.8%) received combination anthracycline and taxane chemotherapy, with mastectomy (98.1%) the most frequent operation. Before NAC, mean tumor size was 4.7 ± 2.9 cm, the most common tumor size was 2–5 cm (66.1%), followed by >5 cm (27.5%), and ≤2 cm (6.4%). Baseline nodal status before NAC was positive and negative in 69.7 and 30.3% of patients, respectively. After NAC, mean residual tumor size was 2.9 ± 2.1 cm, and the most common tumor size was ≤2 cm (47.7%), followed by 2–5 cm (40.4%), and >5 cm (11.9%). Nodal status after NAC was positive and negative in 59.6 and 40.4% of patients, respectively. Before NAC, mean Ki67 index was 36.8% ± 22.5%, while after NAC the corresponding value was 30.6% ± 20.9%. Relative to baseline status, 53 patients (48.6%) had a decreased Ki67 index after NAC and high RD TIL levels (≥30%) were observed in 54 patients (49.5%).

**Table 1:** Clinicopathological characteristics of patients (n = 109).

Characteristics	Mean ± SD	N (%)
**Patients**		109 (100)
**Age at diagnosis (year)**	47.8 ± 10.5	
≤50		73 (67.0)
>50		36 (33.0)
**Menopausal status**		
Pre/peri		68 (62.4)
Post		41 (37.6)
**Histologic subtype**		
Ductal		99 (90.8)
Lobular		3 (2.8)
Others*		7 (6.4)
**Grade**		
I		1 (0.9)
II		58 (53.2)
III		35 (32.1)
Unknown		15 (13.8)
**Neoadjuvant therapy**		
Anthracycline plus taxane		99 (90.8)
Taxane-based		4 (3.7)
Anthracycline-based		6 (5.5)
**Surgery**		
Mastectomy		107 (98.1)
Conservative surgery		2 (1.9)
**Tumor size before NAC (cm)**	4.7 ± 2.9	
≤2		7 (6.4)
2–5		72 (66.1)
>5		30 (27.5)
**Nodal status before NAC**		
Positive		76 (69.7)
Negative		33 (30.3)
**Ki67 before NAC (%)**	36.8 ± 22.5	
<14		17 (15.6)
14–30		44 (40.4)
>30		48 (44.0)
**Residual tumor size (cm)**	2.9 ± 2.1	
≤2		52 (47.7)
2–5		44 (40.4)
>5		13 (11.9)
**Nodal status after NAC**		
Positive		65 (59.6)
Negative		44 (40.4)
**Ki67 after NAC (%)**	30.6 ± 20.9	
<14		35 (32.1)
14–30		29 (26.6)
>30		45 (41.3)
**Ki67 status**		
Decrease		53 (48.6)
No decrease		56 (51.4)
**RD TILs level**		
Low		55 (50.5)
High		54 (49.5)

*Other histological types and distribution were as follows: two medullary carcinoma; two metaplastic carcinoma; one invasive carcinoma with apocrine differentiated carcinoma; one sarcomatous carcinoma; one pleotypic carcinoma. SD, standard deviation; NAC, neoadjuvant chemotherapy; RD, residual disease; TILs, tumor-infiltrating lymphocytes.

During a median follow-up period of 51 months (range, 1 to 97 months) for RFS and 54 months (range, 4 to 101 months) for OS, there were 48 RFS events and 43 deaths. The 3-year RFS and OS rates were 69.7 and 72.0%, respectively. Bias due to loss during follow-up represented 8.26% (nine patients).

### Associations of Changes in Ki67 Index and RD TIL Levels With Clinicopathological Characteristics

Relative to baseline status, 53 patients (48.6%) had a decreased Ki67 index after NAC. We compared the clinicopathological features of patient groups with Ki67 decrease and no Ki67 decrease using the chi-square and Fisher’s exact tests. No differences were identified in age, menopausal status, histological subtype, histological grade, residual tumor size, nodal status after NAC, or RD TIL levels (all P >0.05; **Table 2**).

**Table 2:** The relationship between Ki67 status and other factors.

Characteristics	Ki67 status		P
	Decrease (n = 53)	No decrease (n = 56)	
**Age at diagnosis (year)**			0.840
≤50	35 (66.0)	38 (67.9)	
>50	18 (34.0)	18 (32.1)	
**Menopausal status**			0.711
Pre/peri	34 (64.2)	34 (60.7)	
Post	19 (35.8)	22 (39.3)	
**Histologic subtype**			0.927
IDC	48 (90.6)	51 (91.1)	
No IDC	5 (9.4)	5 (8.9)	
**Grade**			0.380
I–II	30 (56.6)	29 (51.8)	
III	14 (26.4)	21 (37.5)	
Unknown	9 (17.0)	6 (10.7)	
**Residual tumor size (cm)**			0.571
≤2	28 (52.8)	24 (42.9)	
2–5	19 (35.8)	25 (44.6)	
>5	6 (11.3)	7 (12.5)	
**Nodal status after NAC**			0.531
Positive	16 (29.1)	28 (51.9)	
Negative	39 (70.9)	26 (48.1)	
**RD TILs level**			0.504
Low	25 (47.2)	30 (53.6)	
High	28 (52.8)	26 (46.4)	

IDC, invasive ductal carcinoma; NAC, neoadjuvant chemotherapy; RD, residual disease; TILs, tumor-infiltrating lymphocytes.

RD TIL levels were evaluated based on examination of H&E-stained specimens (**Figure 2**). High RD TIL levels (≥30%) were detected in 49.5% of cases. Relationships between RD TIL level (low or high) and clinicopathological characteristics were assessed using chi-square and Fisher’s exact tests (**Table 3**). High RD TIL levels were significantly associated with residual tumor size ≤2 cm (P = 0.049) and negative nodal status after NAC (P = 0.015). No associations were detected with age, menopausal status, histologic subtype, histological grade, or change in Ki67 index (all P >0.05).

**Table 3:** The relationship between TILs and other factors.

Characteristics	RD TILs level		P
	Low (n = 55)	High (n = 54)	
**Age at diagnosis (year)**			0.734
≤50	36 (65.5)	37 (68.5)	
>50	19 (34.5)	17 (31.5)	
**Menopausal status**			0.786
Pre/peri	35 (63.6)	33 (61.1)	
Post	20 (36.4)	21 (38.9)	
**Histologic subtype**			0.742
IDC	49 (89.1)	50 (92.6)	
No IDC	6 (10.9)	4 (7.4)	
**Grade**			0.792
I–II	28 (50.9)	31 (57.4)	
III	19 (34.5)	16 (29.6)	
Unknown	8 (14.5)	7 (13.0)	
**Residual tumor size (cm)**			0.049
≤2	21 (38.2)	31 (57.4)	
2–5	24 (43.6)	20 (37.0)	
>5	10 (18.2)	3 (5.6)	
**Nodal status after NAC**			0.015
Positive	16 (29.1)	28 (51.9)	
Negative	39 (70.9)	26 (48.1)	
**Ki67 status**			0.504
Decrease	25 (45.5)	28 (51.9)	
No decrease	30 (54.5)	26 (48.1)	

TILs, tumor-infiltrating lymphocytes; RD, residual disease; IDC, invasive ductal carcinoma; NAC, neoadjuvant chemotherapy.

### Association of Changes in Ki67 Index and RD TIL Levels With Prognosis

Univariate analyses indicated that there were no significant associations of age, menopausal status, histological subtype, histological grade, or residual tumor size with RFS or OS (all P >0.05; **Table 4**). Taking into consideration clinical practice and statistical power, residual tumor size, nodal status after NAC, RD TIL levels, and Ki67 status were included in multivariate Cox proportional hazard regression models for RFS and OS. On multivariate analyses, no Ki67 decrease status, low RD TIL levels, and positive nodal status after NAC were significantly associated with reduced RFS, with estimated HR values of 2.038 (95% CI: 1.135–3.658, P = 0.017), 2.493 (95% CI: 1.335–4.653, P = 0.004), and 3.207 (95% CI: 1.574–6.535, P = 0.001), respectively (**Table 4**). Moreover, no Ki67 decrease status, low RD TIL levels, and positive nodal status after NAC were also significantly associated with reduced OS, with estimated HR values of 2.187 (95% CI: 1.173–4.077, P = 0.014), 2.499 (95% CI: 1.285–4.858, P = 0.007), and 3.842 (95% CI: 1.756–8.408, P = 0.001), respectively (**Table 4**).

**Table 4:** Univariate and multivariate analyses for RFS and OS in all TNBC patients.

Factor	RFS						OS					
	Univariate analysis			Multivariate analysis			Univariate analysis			Multivariate analysis		
	HR	95% CI	P	HR	95% CI	P	HR	95% CI	P	HR	95% CI	P
**Age at diagnosis (year)**			0.831						0.780			
≤50	1						1					
>50	0.937	0.514–1.707					1.094	0.583–2.052				
**Menopausal status**			0.843						0.783			
Pre/peri	1						1					
Post	0.942	0.525–1.691					1.090	0.591–2.009				
**Histologic subtype**			0.796						0.509			
IDC	1						1					
No IDC	1.130	0.447–2.854					1.370	0.538–3.484				
**Grade**												
I–II	1						1					
III	1.475	0.791–2.748	0.221				1.544	0.799–2.982	0.196			
Unknown	1.150	0.495–2.670	0.745				1.147	0.465–2.834	0.766			
**Residual tumor size (cm)**			0.237			0.739			0.121			0.331
≤2	1						1					
>2	1.414	0.796–2.511					1.625	0.880–3.001				
**Nodal status after NAC**			<0.001			0.001			<0.001			0.001
Negative	1			1			1			1		
Positive	3.739	1.868–7.527		3.207	1.574–6.535		4.503	2.079–9.754		3.842	1.756–8.408	
**Ki67 status**			0.029			0.017			0.018			0.014
Decrease	1			1			1			1		
No decrease	1.910	1.069–3.413		2.038	1.135–3.658		2.114	1.138–3.928		2.187	1.173–4.077	
**RD TILs level**			<0.001			0.004			0.001			0.007
High	1			1			1			1		
Low	2.974	1.612–5.487		2.493	1.335–4.653		3.060	1.592–5.882		2.499	1.285–4.858	

RFS, recurrence-free survival; OS, overall survival; HR, hazard ratio; CI, confidence interval; IDC, invasive ductal carcinoma; NAC, neoadjuvant chemotherapy; RD, residual disease; TILs, tumor-infiltrating lymphocytes.

**Figure 3:**
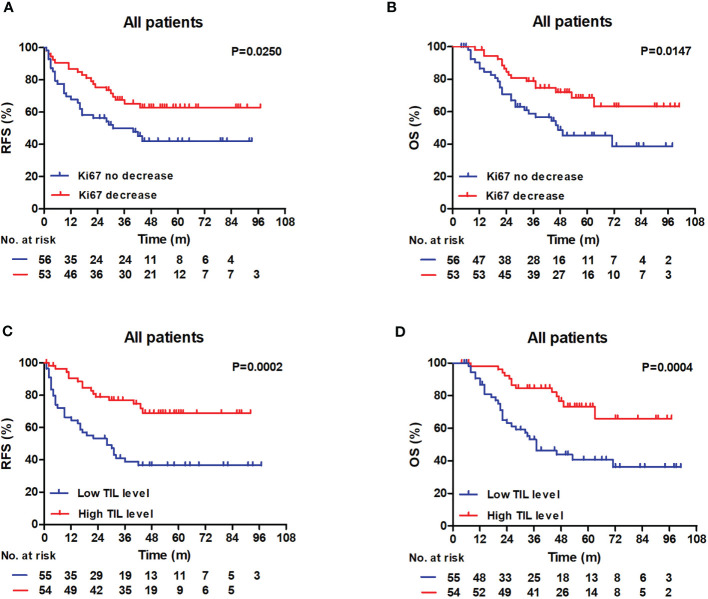
RFS and OS outcomes in the whole patient cohort according to the TIL level and the Ki67 status. **(A, B)** Patients with decreased Ki67 status exhibited raised 3-year RFS and 3-year OS compared with patients without (RFS: 62.8% vs 47.7%, log-rank, P = 0.0250; OS: 78.9% vs 58.8%, log-rank, P = 0.0147). **(C, D)** Patients with low RD TIL levels exhibited reduced 3-year RFS and 3-year OS compared with patients with high RD TIL levels (RFS: 41.1% vs 68.8%, log-rank, P = 0.0002; OS: 53.1% vs 84.6%, log-rank, P = 0.0004). RFS, recurrence-free survival; OS, overall survival; TIL, tumor-infiltrating lymphocyte.

Patients with decreased Ki67 status had higher 3-year RFS and OS rates compared with patients with no Ki67 decrease (RFS: 62.8% vs 47.7%, log-rank P = 0.0250; OS: 78.9% vs 58.8%, log-rank P = 0.0147) (**Figures 3A, B**). In addition, patients with low RD TIL levels exhibited reduced 3-year RFS and OS relative to those with high RD TIL levels (RFS: 41.1% vs 68.8%, log-rank, P = 0.0002; OS: 53.1% vs 84.6%, log-rank, P = 0.0004) (**Figures 3C, D**).

### Prognostic Value of RD TIL Levels According to Ki67 Index Status

In univariate analyses there were no significant associations of age, menopausal status, histological subtype, histological grade, or residual tumor size with RFS or OS in either the Ki67 decrease or no decrease groups (all P >0.05; **Tables 5**, **6**). Taking into consideration clinical practice and statistical power, residual tumor size, nodal status after NAC, and RD TIL levels were included in multivariate Cox proportional hazard regression models. In the no Ki67 decrease group, low RD TIL levels were significantly associated with reduced RFS and OS, with estimated HR values of 3.567 (95% CI: 1.475–8.624, P = 0.005) and 3.873 (95% CI: 1.512–9.918, P = 0.005), respectively. Moreover, positive nodal status after NAC was significantly associated with reduced RFS and OS, with estimated HR values of 2.955 (95% CI: 1.167–7.481, P = 0.022) and 3.335 (95% CI: 1.227–9.068, P = 0.018), respectively (**Table 5**); however, in the Ki67 decreased group, only positive nodal status after NAC was associated with reduced RFS and OS, with estimated HR values of 3.517 (95% CI: 1.165–10.614, P = 0.026) and 4.391 (95% CI: 1.241–15.534, P = 0.022), respectively (**Table 6**).

**Table 5:** Univariate and multivariate analyses for RFS and OS in Ki67 no decrease group.

Factor	RFS						OS					
	Univariate analysis			Multivariate analysis			Univariate analysis			Multivariate analysis		
	HR	95% CI	P	HR	95% CI	P	HR	95% CI	P	HR	95% CI	P
**Age at diagnosis (year)**			0.716						0.786			
≤50	1						1					
>50	0.864	0.393–1.899					1.118	0.500–2.500				
**Menopausal status**			0.690						0.942			
Pre/peri	1						1					
Post	0.858	0.405–1.819					1.029	0.476–2.223				
**Histologic subtype**			0.801						0.957			
IDC	1						1					
No IDC	0.857	0.259–2.841					0.967	0.290–3.221				
**Grade**												
I–II	1						1					
III	1.177	0.540–2.565	0.682				1.225	0.544–2.760	0.624			
Unknown	0.722	0.209–2.503	0.608				0.854	0.245–2.981	0.805			
**Residual tumor size (cm)**			0.664			0.441			0.816			0.950
≤2	1						1					
>2	0.850	0.409–1.769					1.096	0.508–2.366				
**Nodal status after NAC**			0.003			0.022			0.002			0.018
Negative	1			1			1			1		
Positive	4.020	1.623–9.957		2.955	1.167–7.481		4.641	1.746–12.339		3.335	1.227–9.068	
**RD TILs level**			0.001			0.005			0.001			0.005
High	1			1			1			1		
Low	4.577	1.932–10.841		3.567	1.475–8.624		5.093	2.033–12.755		3.873	1.512–9.918	

RFS, recurrence-free survival; OS, overall survival; HR, hazard ratio; CI, confidence interval; IDC, invasive ductal carcinoma; NAC, neoadjuvant chemotherapy; RD, residual disease; TILs, tumor-infiltrating lymphocytes.

**Table 6:** Univariate and multivariate analyses for RFS and OS in Ki67 decrease group.

Factor	RFS						OS					
	Univariate analysis			Multivariate analysis			Univariate analysis			Multivariate analysis		
	HR	95% CI	P	HR	95% CI	P	HR	95% CI	P	HR	95% CI	P
**Age at diagnosis (year)**			0.873						0.817			
≤50	1						1					
>50	1.079	0.425–2.742					1.127	0.409–3.104				
**Menopausal status**			0.995						0.900			
Pre/peri	1						1					
Post	1.003	0.395–2.549					1.067	0.387–2.943				
**Histologic subtype**			0.558						0.314			
IDC	1						1					
No IDC	1.550	0.357–6.721					2.151	0.484–9.555				
**Grade**												
I–II	1						1					
III	1.739	0.618–4.895	0.295				1.889	0.609–5.862	0.271			
Unknown	1.935	0.595–6.290	0.273				1.755	0.462–6.669	0.409			
**Residual tumor size (cm)**			0.064			0.055			0.093			0.079
≤2	1						1					
>2	2.420	0.950–6.161					2.391	0.865–6.611				
**Nodal status after NAC**			0.026			0.026			0.022			0.022
Negative	1			1			1			1		
Positive	3.517	1.165–10.614		3.517	1.165–10.614		4.391	1.241–15.534		4.391	1.241–15.534	
**RD TILs level**			0.248			0.397			0.350			0.457
High	1						1					
Low	1.711	0.688–4.257					1.602	0.596–4.309				

RFS, recurrence-free survival; OS, overall survival; HR, hazard ratio; CI, confidence interval; IDC, invasive ductal carcinoma; NAC, neoadjuvant chemotherapy; RD, residual disease; TILs, tumor-infiltrating lymphocytes.

**Figure 4:**
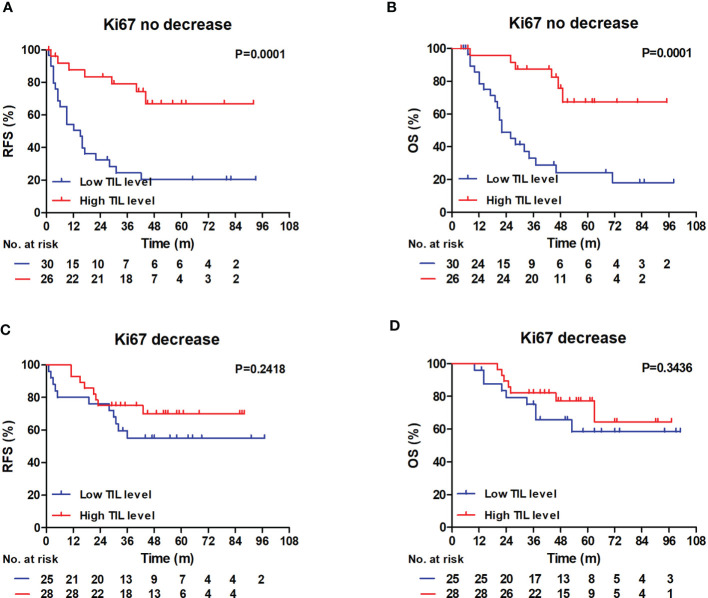
RFS and OS outcomes in the Ki67 no decrease and decrease groups according to the TIL level. **(A, B)** In Ki67 no decrease group, Patients with low RD TIL levels exhibited reduced 3-year RFS and 3-year OS compared with patients with high RD TIL levels (RFS: 24.4% *vs* 79.1%, log-rank, P = 0.0001; OS: 33.1% *vs* 87.5%, log-rank, P = 0.0001). **(C, D)** In Ki67 decrease group, no significant differences were showed in RFS and OS graphs (RFS: log-rank, P = 0.2418; OS: log-rank, P = 0.3436). RFS, recurrence-free survival; OS, overall survival; TIL, tumor-infiltrating lymphocyte.

In Kaplan–Meier analyses, patients with high RD TIL levels had significantly better RFS and OS rates than those with low RD TIL levels in the no Ki67 decrease group (RFS: log-rank P = 0.0001; OS: log-rank P = 0.0001) (**Figures 4A, B**). The differences in 3-year RFS and OS between patients with low or high RD TIL levels were 24.4% vs 79.1% and 33.1% vs 87.5%, respectively; however, in the Ki67 decrease group, no significant differences in RFS or OS were detected (RFS: log-rank P = 0.2318; OS: log-rank P = 0.3436) (**Figures 4C, D**).

## Discussion

In this study, we examined 109 patients with TNBC who did not achieve pCR after NAC, to investigate the prognostic significance of changes in Ki67 index and RD TIL levels. We found that no Ki67 decrease status and low RD TIL levels after NAC were significantly associated with worse RFS and OS in patients with TNBC and RD. Moreover, the magnitude of the prognostic value of RD TIL levels differed according to Ki67 status, with the greatest absolute differences observed in patients with no decrease in Ki67 index.

TIL levels are associated with TNBC patient prognosis, with high TIL levels linked to better treatment response and clinical outcomes in both neoadjuvant (9, 26, 27) and adjuvant (28–30) settings. In a retrospective study involving 375 TNBC RD cases, Luen et al. (13) reported that RD TIL levels provided independent and additional prognostic information beyond pre-treatment TIL levels in patients with primary TNBC treated with NAC, for both RFS (χ^2^ 9.88, P = 0.002) and OS (χ^2^ 8.02, P = 0.005). These findings were supported by other studies (12, 31). In this study we focused on the prognostic impact of RD TIL levels in patients with TNBC treated with NAC. Our results show that low (<30%) RD TIL levels are an independent prognostic factor associated with reduced RFS and OS in patients with primary TNBC, with estimated HR values of 2.493 (95% CI: 1.335–4.653, P = 0.004) and 2.499 (95% CI: 1.285–4.858, P = 0.007), respectively, consistent with published studies (12, 13). Moreover, we found that low RD TIL levels were associated with larger residual tumor size and positive nodal status. Overall, evidence indicates that low RD TIL levels are associated with more aggressive tumors, possibly because RD TIL levels are directly related to the magnitude of host anti-tumor adaptive immune responses following NAC (13).

Many previous investigations have shown that breast cancer Ki67 index status changes after NAC (15, 16). In this study, we also detected differences in Ki67 index before and after NAC; 48.6% of residual tumors exhibited a decrease in Ki67 index after NAC. Furthermore, we explored the correlation between pre-NAC Ki67 index and Ki67 changes after NAC and found that decreased Ki67 status was related to high Ki67 index before NAC (**Table S1**; P = 0.014). Decreased Ki67 index after NAC has been reported as significantly associated with better prognosis in patients with breast cancer (16, 18, 32); however, prognostic information regarding Ki67 changes is limited in patients with TNBC and RD, and other studies (17, 18) showed that decreased Ki67 expression after NAC had clear prognostic significance in patients with TNBC and RD although, unfortunately, they did not provide the results of multivariate analysis of the TNBC group. Hence, our study may provide some new information. During median follow-up periods of 51 and 54 months for RFS and OS, respectively, we found that no Ki67 decrease status after NAC was significantly associated with worse RFS and OS in patients with TNBC and RD. In multivariate Cox analyses, no Ki67 decrease status was significantly associated with reduced RFS (HR: 2.038, 95% CI: 1.135–3.658, P = 0.017) and OS (HR: 2.187, 95% CI: 1.173–4.077, P = 0.014). In Kaplan–Meier analyses, patients with decreased Ki67 status had higher 3-year RFS and OS rates than patients without (RFS: 62.8% vs 47.7%, log-rank P = 0.0250; OS: 78.9% vs 58.8%, log-rank P = 0.0147). In contrast, a retrospective study of 435 patients with breast cancer who did not achieve pCR after standard NAC with anthracycline and paclitaxel reported no prognostic significance of Ki67 changes in the TNBC group (32); the difference between these findings and our data may be due to differences in sample source and the definition of Ki67 decrease.

Interestingly, in this investigation we observed that the prognostic significance of RD TIL levels differed markedly according to Ki67 status in patients with TNBC who received NAC. In the no Ki67 decrease group, low RD TIL levels were significantly associated with reduced RFS and OS, with estimated HR values of 2.733 (95% CI: 1.122–6.658, P = 0.027) and 4.114 (95% CI: 1.335–12.673, P = 0.014), respectively; however, this is in contrast with the lack of significant prognostic influence of RD TIL levels in the Ki67 decrease group (P >0.05). The relationship between Ki67 changes and RD TIL levels remains somewhat unclear. We explored the correlation between RD TIL level and Ki67 status after NAC, and found that there was no significant statistical correlation between the two factors (**Table 3**; P = 0.504). A larger patient sample may be required to further explore this correlation. In addition, we found that RD TIL level had stronger prognostic significance in the no Ki67 decrease group. We suspect that this finding may be related to changes in tumor proliferation and the tumor microenvironment that occur after NAC. TIL levels reflect the tumor immune microenvironment, and high TIL levels in RD may indicate a strong anti-tumor immune response after NAC. Ki67 index reflects the ability of tumor cells to proliferate. No decrease in Ki67 status after NAC may reflect a limited effect of NAC on tumor proliferative capacity and activity, and the observed prognostic correlation with high RD TIL levels is logical in this context. Increased understanding of the interactions between cancer cell proliferation regulation and tumor immune responses may advance treatment of TNBC in the future.

This study has some limitations. First, the sample size was small and patients were recruited from a single center; therefore, selection bias was unavoidable. Second, due to the amount of work and the retrospective nature of this study, we evaluated RD based on tumor size and nodal status, which is less robust than modern methods, such as residual cancer burden index. Third, in our study cohort, four patients had a potential follow-up period of <36 months (range, 30–35 months). Therefore, a large-scale, multi-center, prospective validation study, with longer follow-up period is needed to further clarify the results of this study.

## Conclusion

In summary, in this study we found that decreased Ki67 index and high RD TIL levels were associated with superior RFS and OS in patients with primary TNBC and RD following NAC. Larger positive effects of TILs on RFS and OS were observed in patients with no Ki67 decrease status. Hence, assessment of Ki67 index changes and RD TIL levels after NAC could provide valuable prognostic information for patients with TNBC.

## Data Availability Statement

The raw data supporting the conclusions of this article will be made available by the authors, without undue reservation.

## Ethics Statement

The studies involving human participants were reviewed and approved by the Ethics Committee of the First Affiliated Hospital of Chongqing Medical University. Written informed consent for participation was not required for this study in accordance with the national legislation and the institutional requirements.

## Author Contributions

YiW was responsible for original conception and design, analysis of data, search of the literature, correction, and editorship of the manuscript. BZ was responsible for original conception, acquisition of clinical data, correction, and English editing. YY was responsible for acquisition and re-evaluation of pathological sections, analysis of pathological data, and search of the literature. YuW, RC, ZT, and MH were responsible for acquisition of clinical data, correction, and English editing. SL was responsible for design, English editing, correction, and approval of the final version. All authors contributed to the article and approved the submitted version.

## Funding

This work was supported by the National Natural Science Foundation of China under Grant number 81272265.

## Conflict of Interest

The authors declare that the research was conducted in the absence of any commercial or financial relationships that could be construed as a potential conflict of interest.
