# Supplementary material for: Ki67 Index Changes and Tumor-Infiltrating Lymphocyte Levels Impact the Prognosis of Triple-Negative Breast Cancer Patients With Residual Disease After Neoadjuvant Chemotherapy
Source: Front Oncol. 2021 Jun 21;11:668610. doi: 10.3389/fonc.2021.668610 (PMC8256666; doi:10.3389/fonc.2021.668610)
Supplement: Supplementary file 1 [file Table_1.docx]

**Table S1** The relationship between Ki67 status and Ki67 index before NAC

| **Characteristics** | **Ki67 status** | | **P** |
| --- | --- | --- | --- |
|  | **Decrease (n=53)** | **No decrease (n=56)** |  |
| **Ki67 index before NAC (%)** | |  | 0.014 |
| ≤ 14 | 4 (7.5) | 13 (23.2) |  |
| 14-30 | 19 (21.4) | 25 (44.6) |  |
| ＞ 30 | 30 (56.6) | 18 (32.1) |  |

**Abbreviations:** NAC, neoadjuvant chemotherapy.
